# Unlocking the plant growth-promoting potential of yeast spp.: exploring species from the Moroccan extremophilic environment for enhanced plant growth and sustainable farming

**DOI:** 10.1093/femsle/fnae015

**Published:** 2024-02-28

**Authors:** Anas Raklami, Olubukola Oluranti Babalola, Martin Jemo, Ahmed Nafis

**Affiliations:** AgroBiosciences Program, College of Sustainable Agriculture and Environmental Sciences (CAES), Mohammed VI Polytechnic University (UM6P), Lot 660, Hay Moulay Rachid, Benguerir 43150, Morocco; Food Security and Safety Focus Area, Faculty of Natural and Agricultural Sciences, North-West University, Private Bag X2046, Mmabatho 2735, South Africa; AgroBiosciences Program, College of Sustainable Agriculture and Environmental Sciences (CAES), Mohammed VI Polytechnic University (UM6P), Lot 660, Hay Moulay Rachid, Benguerir 43150, Morocco; Microbiology and Antimicrobial Agents Team, Laboratory of Plant Biotechnology, Ecology and Valorization of Ecosystems (LB2VE/URL-CNRST n°10), Faculty of Sciences, Chouaïb Doukkali University, El Jadida 24000, Morocco

**Keywords:** extreme Moroccan environment, rare yeast, stress abiotic resistance, plant growth-promoting, *Medicago sativa*, biofertilizer

## Abstract

In this study, we successfully isolated two distinct yeasts from Moroccan extreme environments. These yeasts were subjected to molecular characterization by analyzing their Internal Transcribed spacer (ITS) regions. Our research thoroughly characterizes plant growth-promoting abilities and their drought and salt stress tolerance. In a greenhouse assay, we examined the impact of selected yeasts on *Medicago sativa*’s growth. Four treatments were employed: (i) control without inoculation (NI), (ii) inoculation with L1, (iii) inoculation with L2, and (iv) inoculation with the mixture L1 + L2. L1 isolated from Toubkal Mountain shared 99.83% sequence similarity to *Rhodotorula mucilaginosa*. Meanwhile, L2, thriving in the arid Merzouga desert, displayed a similar identity to *Naganishia albida* (99.84%). Yeast strains were tolerant to NaCl (2 M) and 60% PEG (polyethylene glycol P6000) in case of drought. Both strains could solubilize phsphorus, with L2 additionally demonstrating potassium solubilization. In addition, both strains produce indole acetic acid (up to 135 µl ml^−1^), have siderophore ability, and produce aminocyclopropane-1-carboxylic acid deaminase. Isolates L1 and L2, and their consortium showed that the single or combined strain inoculation of *M. sativa* improved plant growth, development, and nutrient assimilation. These findings pave the way for harnessing yeast-based solutions in agricultural practices, contributing to enhanced crop productivity and environmental sustainability.

## Teaser

One-sentence summary: Isolate non-*Saccharomyces* yeast resident of Moroccan extreme environments, study their plant growth promotion traits, and evaluate their usefulness to promote plant growth for sustainable agriculture.

## Introduction

Extreme environments (EEs) host an extensive array of microbial species, many interacting mutualistically with plants, and previous studies have characterized these extremophilic microbe–plant interactions (Nafis et al. 2018, 2019, Devi et al. 2020, Tapia-Vázquez et al. 2020). Lately, there has been a surge of interest in the isolation, identification, and exploitation of extremophile microbes. Their distinctive abilities, metabolic capabilities, and activities have made them exceptionally appealing to industries across diverse biotechnological fields (Tsuji et al. 2018, Tapia-Vázquez et al. 2020, Loeto et al. 2021, Martínez-Ávila et al. 2021). For instance, Tapia-Vázquez et al. (2020) isolated and characterized four yeast strains from rhizospheric soil collected from the Xinantécatl volcano in Mexico. Their research highlighted the plant growth-promoting (PGP) abilities of these extremophilic yeasts, which further reinforced the significance of such microbial resources in sustainable agriculture.

Considerable research efforts have been directed toward using specific microorganisms, mainly plant growth-promoting rhizobacteria (PGPR), within agriculture. These PGPR serve as biofertilizers or biostimulants, contributing to improved soil fertility, enhanced plant growth, increased yield, and, ultimately, a better use of chemical fertilizer under diverse environmental conditions (Raklami et al. 2019, Anli et al. 2020, Mokabel et al. 2022, Sedri et al. 2022). Yeasts have received less attention among microbial species due to their relatively low population density. Astonishingly, even to this day, <1% of the yeast species in nature have been isolated, identified, and discovered (Kurtzman and Piškur 2006, Starmer and Lachance 2011, Segal-Kischinevzky et al. 2022).

As a prominent eukaryotic organism, yeast is renowned for its extraordinary biodiversity and finds widespread utility in various biotechnological applications, including agriculture (Amprayn et al. 2012, Nandy and Srivastava 2018). A key aspect of their positive impact could be attributed to their PGP properties, including phytohormones (Ignatova et al. 2015, Kumla et al. 2020), phosphate, potassium, and zinc solubilization (Mirabal Alonso et al. 2008, Tapia-Vázquez et al. 2020, Srinivasan et al. 2022), nitrogen fixation, sulfur oxidation (Srinivasan et al. 2022), and siderophore production (Sansone et al. 2005, Silva et al. 2020, Tapia-Vázquez et al. 2020). Furthermore, certain species were recognized for their exopolysaccharide secretion (Hamidi et al. 2020), accelerating the decomposition of organic materials into usable nutrients, enhancing soil water-holding capacity, and improving the overall soil health (Ramya et al. 2021). Additionally, yeasts release a spectrum of bioactive compounds, including vitamins, hormones, and enzymes. These are vital in stimulating plant growth, managing soil-borne pathogens, and making the crop stress tolerant (Tsuji et al. 2018, Loeto et al. 2021, Srinivasan et al. 2022). These characteristics are paramount in sustainable agriculture, as they are firmly rooted in biological processes that actively bolster plant growth and productivity. Moreover, they contribute to the maintenance of soil fertility. Harnessing these attributes of extremophilic yeast presents a promising avenue for enhancing agricultural sustainability, reducing the reliance on chemical inputs, and nurturing the long-term health of our soils. Some species belonging to the genera *Dothideomycetes, Pseudozyma, Sporidiobolus, Hanseniaspora*, and *Candida* were able to promote plant growth (Amprayn et al. 2012, Fu et al. 2016, Jaiboon et al. 2016, Fernandez-San Millan et al. 2020).

While widespread studies have propagated arbuscular mycorrhizal fungi and PGPR in various agricultural contexts, as exemplified by studies such as those by Uzoh and Babalola (2018), Igiehon and Babalola (2021), Agbodjato et al. (2022), Xin et al. (2022), and Raklami et al. (2023), the potential to use yeasts as PGP agents remain underexploited. Hence, using yeasts, especially the non-*Saccharomyces* species, represents a poorly explored field with great potential. This offers a promising and sustainable agriculture solution to combat nutrient deficiencies. Notably, despite a few existing reports on PGP yeast, our study marks the pioneering effort in exploring Moroccan yeast biodiversity and evaluating their impact on plant growth.

The primary objective of our study was to isolate and characterize the growth-promoting stress-tolerant yeast isolated from two unexplored Moroccan extreme environments (MEEs). These environments are characterized by long-term harsh conditions. Specially, we aimed at the following:

1. Isolate non-*Saccharomyces* yeast and comprehensively characterize the stress-tolerant yeast strains in these EEs.
2. Assess and compare the plant growth-promoting traits of these isolated yeast strains.
3. Determine the parctical benefits of these yeast strains in stimulating the growth of *Medicago sativa*, a important agricultural crop.

## Materials and methods

### Moroccan extremophilic sites description and soil sampling

Soil samples were procured from two distinct geographical locations, the Toubkal Mountain (TM) and the Merzouga desert (MD). Toubkal is a mountain summit in southwest Morocco in the high Moroccan Atlas Mountains at the Toubkal National Park. At 4167 m, TM is the highest peak in Morocco, North Africa, and the entire Arab-speaking world (Vieira et al. 2017). The region is characterized by a notable annual rainfall of >600 mm, primarily attributed to convective summer rains. However, it is also known for its low temperatures, with the average maximum temperature during the warmest month of the year being 16°C and the average minimum temperature during the coldest month plunging to −10.5°C. Snowfall is expected from November through April or May (Vieira et al. 2017). Merzouga, a small village in southeastern Morocco, is renowned for its proximity to the Erg Chebbi, the most significant in the country, which features Morocco’s highest dune. The climate in Merzouga is characterized by aridity, with an average annual precipitation of 85 mm. The region experiences a dry, scorching summer, with daytime temperatures often surpassing/frequently exceeding 40°C, contrasted by freezing evenings. The area is known for low water activity and intense radiation (Gommeaux et al. 2010, Manni and Filali-Maltouf 2022).

### Isolation of the stress-tolerant yeasts

The two used yeasts were isolated from soil collected from distinct parts of the TM region (31.05917′′N–7.91583′′W) (for isolate L1) and the desert soil from Merzouga (31.147643′′N, −3.974280′′W) (for isolate L2). The isolation was performed using the standard method. Ten grams of Toubkal and Mezrouga soils were meticulously homogenized in 90 ml of sterile physiological water (PW). Subsequently, we prepared dilutions of each soil using the serial 10-fold dilution technique. Then, 0.1 ml from each dilution was spread onto Sabouraud agar supplemented with chloramphenicol (100 mg ml^−1^) to prevent the growth of bacteria. The Petri dishes were incubated at 30°C for at least one week. The developed colonies were isolated and purified on the same medium and stored in 25% (w/v) glycerol at −20°C for subsequent investigations.

### Identification of cultivable yeast

Molecular identification of the internal transcribed spacer (ITS) region of each of the isolated strains was performed using the forward primer ITS1 (5′-TCCGTAGGTGAACCTGCGG-3′) and the reverse primer ITS4 (5′-TCCTCCGCTTATTGATATGC-3′) and sequenced at Macrogen company, Korea (http://dna.macrogen.com). The ITS region, situated between the 18S and 5.8S rRNA region genes in the eukaryotic ribosomal RNA gene cluster, tends to exhibit sufficient genetic variability among different yeast species, making it a valuable target for species-level identification. The sequences obtained were classified using an essential local alignment search tool (BLAST) to compare our sequences with the deposited copies in the National Center for Biotechnology Information (NCBI eucaryotic database) (Kim et al. 2012). The sequences were aligned in the Molecular Evolution Genetics Analysis (MEGA) software (v12.0) using ClustalW (Larkin et al. 2007, Hall 2013). The phylogenetic tree was constructed based on the Tamura–Nei model using neighbor-joining analyses (bootstrap of 1000) (Tamura et al. 2011).

### Characterizing the isolated yeasts from extremophilic environments

#### Screening for salt and osmotic tolerance

The evaluation of salt and osmotic stress tolerance was carried out by monitoring the growth on Sabouraud medium amended with varying concentrations of NaCl (ranging from 0.092 to 4 mM) and polyethylene glycol P6000 (from 1.25% to 60%) using microtiter plates. Subsequently, the plates were incubated at 28°C for 96 h. After incubation, we measured the optical density at 600 nm using a microtiter plate reader.

#### Phosphate and potassium solubilizing capacity

We conducted the phosphate solubilizing activity using NBRIY broth, supplemented with 5 g l^−1^ tricalcium phosphate or Moroccan phosphate rock as the exclusive inorganic phosphate source (Nafis et al. 2019). The medium was inoculated with a 200-µl yeast solution, washed three times with PW, and adjusted to an optical density (OD_600_) of 0.1. The medium was then incubated at 28°C on a rotatory shaker, agitating at 140 rpm, for 96 h. After incubation, the yeast cells were separated by centrifugation (6000 rpm) for 10 min. In the supernatant, we measured the pH and soluble phosphate as described by Nagul et al. (2015).

Potassium solubilization capacity was determined onto Aleksandrov agar medium supplemented with 5 g l^−1^ Mica powder (Meena et al. 2015). The agar plates were inoculated following the drop-on-plate method described by Alikhani et al. (2006) with a bacterial solution washed and adjusted to OD_600_ = 0.8. The plates were incubated for 7 days at 30°C, and the results were displayed as the ratio of the clear zone surrounding the colonies and the diameter of the colony.

#### Indole acetic acid and siderophore production

To evaluate the ability of isolated yeasts to produce indole acetic acid (IAA), strains were inoculated into 100 ml of Luria Bertani broth (Kumla et al. 2020) supplemented with 1.02 g l^−1^ of l-tryptophan as IAA precursor. The yeast cell density was adjusted to 0.8 optical density (OD_600_ = 0.8). After incubation at 28°C on a rotatory shaker, agitating at 140 rpm, 1 ml of the supernatant was mixed with 2 ml of Salkowski’s reagent and two drops of phosphoric acid. This solution was immediately incubated in the dark for 30 min. The intensity of the red color formed was quantitatively determined by measuring the absorbance at 530 nm (Rodrigues et al. 2018). Concerning the siderophore estimation, the test was carried out using the standard Chrome Azurol-S (CAS) test in a solid medium. The siderophore production was indicated by measuring the orange–yellow halo caused by the change of the blue of the CAS medium (Louden et al. 2011).

#### Aminocyclopropane-1-carboxylic acid deaminase production

The isolated yeast strains were evaluated for their capacity to produce aminocyclopropane-1-carboxylic acid (ACC) deaminase on the sterile minimal DF (Dworkin and Foster) salts media (4.0 g KH_2_PO_4_, 6.0 g Na_2_HPO_4_, 0.2 g MgSO_4_·7H_2_O, 2.0 g glucose, 2.0 g gluconic acid, and 2.0 g citric acid with 1 mg FeSO_4_·7H_2_O, 10 mg H_3_BO_3_, 11.19 mg MnSO_4_·H_2_O, 124.6 mg ZnSO_4_·7H_2_O, 78.22 mg CuSO_4_·5H_2_O, 10 mg MoO_3_, pH 7.2) amended with 3 mM ACC instead of (NH_4_)_2_SO_4_ as sole nitrogen source (Dworkin and Foster 1958). The yeast strains were inoculated to minimal DF and incubated at 28°C for 24 h. Then, the ACC deaminase activity was quantified spectrophotometrically by α-ketobutyrate production at 540 nm by comparing with the standard curve of α-ketobutyrate, which ranged from 0.1 to 1.0 µmol (Honma and Smmomura 1978). The protein estimation was conducted using Bradford’s procedure (Bradford 1976). This activity was expressed as nmol of α-ketobutyrate produced per milligram of cellular protein per hour, and it served as a critical indicator of the enzyme’s efficiency.

#### Biological material, experimental design, and plant bioassay

To evaluate the *in-vivo* impact of the isolated strains, we selected *M. sativa* (Demnate landscape variety) as the plant model. The experiment design employed in this study followed a randomized complete block (RCDB) with four treatments (Trt) and four replications, each containing 12 seeds. The first Trt (NI) was the control and involved noninoculated plants, the second Trt (L1) was inoculated with *Rhodotorula* sp. L1, the third Trt (L2) received inoculation with *Naganishia* sp. L2, and the fourth Trt (AM) was inoculated with the mixture of the two strains (L1 + L2). The yeast inoculum was prepared by culturing the selected strains in Sabouraud broth (for 3–4 days at 28°C). Following this incubation, yeast cells were harvested by centrifugation (6000 rpm, 10 min), washed once with sterile distilled water (DW), and then resuspended in DW to the final concentration (OD_600_ = 1). Each pot received 10 ml of the appropriate yeast strains, and the mixed solution was achieved by mixing an equal volume of each strain.

*Medicago sativa* seeds were disinfected by immersion in sodium hypochlorite diluted 1/5 (v/v) for 5 min. Afterward, the seeds were placed on wet filter paper in Petri dishes and germinated in the dark at 25°C for 24 h. Once the seeds sprouted, they were transplanted into 2-l plastic pots filled with a peat and perlite mixture with a 1:1 ratio, which had been previously sterilized. All pots were individually placed within trays in a controlled greenhouse, where they received natural daylight ranging from 250 to 1000 µmol m^2-1^ sec^−1^. The temperature was maintained at 25/21°C day/night with a relative humidity of 40%–60% relative humidity. Plants were irrigated with 250 ml DW twice weekly to ensure regular water availability through treatments.

After 60 days from sowing, both the shoot and roots were harvested. The roots were separated from the shoots and carefully rinsed, with excess water removed using a paper towel. Subsequently, the length of the shoot and root was recorded. The dry weight of the shoots and roots was measured by subjecting them to oven drying for 72 h at 70°C.

Determining mineral elements (N, P, K) was performed after the mineralization of plant materials (shoot). Total nitrogen content was measured according to the method described by Rodier (1984). Phosphorus was determined according to the protocol described by Olsen and Sommers (1982). Meanwhile, K was determined using a flame photometer (AFP 100 flame photometer).

### Statistical analysis

We employed a Statistical Analysis System software for the data analyses, using the JMP module from 2019. One-way analyses of variance (ANOVAs) were conducted to evaluate significant differences among treatments. To compare means and determine specific differences, we applied Tukey’s test method. Significant differences at *P* < 0.05 were denoted by different letters. Values sharing the same letter were considered not significantly different at *P* < 0.05.

## Results

### Physico-chemical characteristics of the soil samples

The soil properties of TM and MD demonstrate distinctive attributes reflecting the diverse environmental conditions present in these two Moroccan sites. In TM, the soil is characterized by a pH level of 7.9, indicating a slightly alkaline environment. The electrical conductivity (EC) is measured at 2.14, suggesting moderate salinity levels. Furthermore, the total organic carbon (TOC) content is 1.02, indicating a relatively enriched organic matter status. Conversely, the sandy soil properties found in the arid expanse of MD differ significantly. The pH is slightly higher at 8.04, reflecting a more alkaline nature than TM. The EC value is lower at 0.95, signaling lower salinity levels. The TOC content is 0.96, indicating a reduced organic matter presence compared to the mountainous counterpart. These variations in soil characteristics highlight the impact of climate, vegetation, and geological factors on the soil composition, illustrating the contrasting EE of the high-altitude TM and the arid MD.

### Identity of cultivable yeast strains

**Figure 1.**
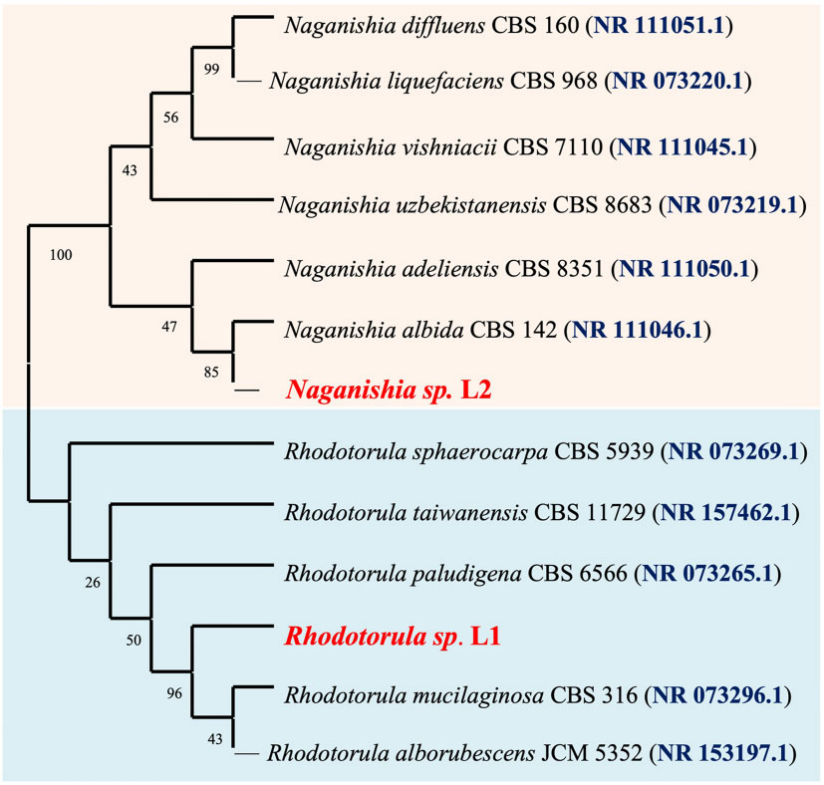
Maximum-likelihood tree based on ITS gene sequence showing the relations between the stress-tolerant yeast strains. The numbers at the nodes indicate the levels of bootstrap support based on maximum-likelihood analyses of 1000 resampled data sets (only values >50% are shown).

Using a culture-dependent method, two yeast strains were isolated, purified, and identified based on ITS sequencing. The BLAST homology search revealed that the red-strain isolate L1 belonged to the genus *Rhodorula* and exhibited a striking 99.83% sequence similarity to *Rhodotorula mucilaginosa*. In contrast, the isolate L2 was assigned to *Naganishia* genus and displayed 99.84% sequence identity with the *Naganishia albida* reference strains. To further understand the evolutionary relationships of the isolated yeasts and their close relatives, we employed the neighbor-joining method based on the ITS1/ITS4 (Fig. 1).

### Characterizing the isolated yeasts from extremophilic environments

#### Screening for salt and osmotic tolerance

We expand our research to evaluate the ability of the extremophilic yeasts to withstand abiotic stress, such as those induced by salt and drought (Table 1). Concerning salt tolerance, all extremophilic yeast showed their high resistance to salt stress and could tolerate 2 M of NaCl (Table 1). Regarding drought tolerance, extremophilic yeast strains could also tolerate 60% of P6000.

**Table 1.** Plant growth-promoting (PGP) traits of the isolated endophytic actinobacterial strains.

Strain	Salt tolerance (M)	Drought tolerance (%P6000)	Tricalcium phosphate solubilization		Rock phosphate solubilization		IAA production (µg/ml)	Potassium solubilization (halo diameter)	Siderophore production (halo diameter)	ACC deaminase activity (µmol/mg protein/h)
			pH	P content (mg/l)	pH	P content (mg/l)				
*Rhodotorula* sp.	2	60	4.40	112.57 ± (3.28)^a^	4.62	170.66 ± (7.19)	134.71 ± (4.76)^a^	0	0.9	2070.65 ± (31.19)^b^
*Naganishia* sp.	2	60	5.69	58.85 ± (3.01)^b^	5.34	181.97± (2.09)	11.38 ± (0.14)^b^	2.5	0.8	2679.97 ± (66.00)^a^

Means (±standard deviation) within the same column followed by different letters are significantly different at *P* < 0.05.

#### Characterizing the isolated yeasts from extremophilic environments

According to the results, the isolated yeast strains demonstrated the ability to solubilize both complex inorganic phosphate, specifically tricalcium and rock phosphate, as shown in Table 1. The solubilization activity of tricalcium phosphate was the most significant in the case of *Rhodotorula* sp. L1, with a solubilization activity of 113 mg ml^−1^. Conversely, for rock phosphate, *Naganishia* sp. L2 exhibited the highest activity (182 mg ml^−1^) (Table 1). The solubilization activity observed in both sources was accompanied by a notable medium acidification, with pH levels around 4–5. This acidification pattern suggests that the solubilization process might be attributed to the production of organic acids. Furthermore, potassium solubilization was recorded in the case of the *Naganishia* sp. L2, with a 2.5 cm halo diameter. The assay for IAA production revealed that both yeast trains could produce IAA using l-tryptophan as a precursor, with a significant production (up to 130 mg l^−1^) observed in the case of *Rhodotorula* sp. L1. Investigation of siderophore production showed that both strains could produce siderophores to chelate iron from the medium. Additionally, *Rhodotorula* sp. L1, and *Naganishia* sp. L2 exhibits ACC deaminase activity, up to 2000 µmol mg protein^−1^ h^−1^ (Table 1). The results of the PGP activities strongly support the potential use of these yeast strains as bioinoculants, particularly in semi-arid regions.

#### The growth-promoting effect of yeast isolates on *Medicago sativa* growth

**Figure 2.**
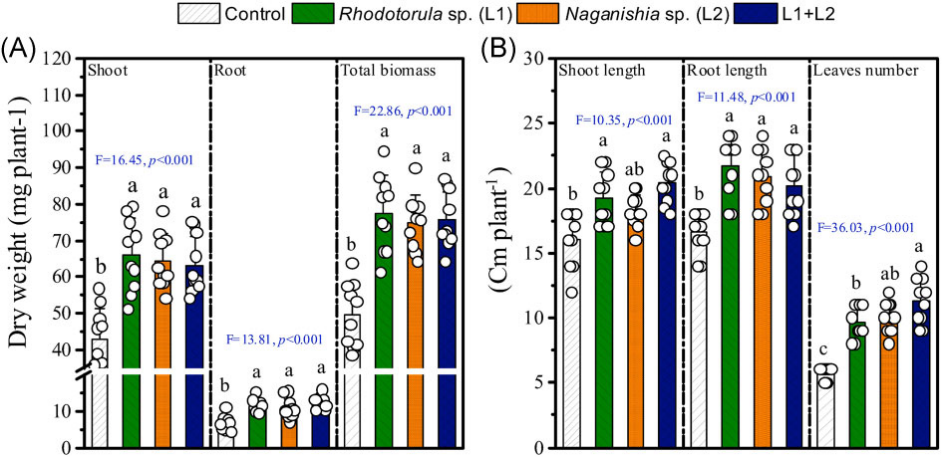
Shoot and root, and total biomass dry weight (A), and lengths of shoot and root, and numbers of leaves (B) of *Medicago sativa* submitted to different treatments [control without inoculation, *Rhodotorula* sp. L1; *Naganishia* sp. L2; and the consortia of A3 + A4]. Means (±SD) within the same parameter followed by different letters are significantly different at *P* < 0.05. Symbols are the number of replicate (10). The Tukey’s test method was used to separate means that were different at *P* ≤ 0.05. Values in columns followed by the same letter are not significantly different at *P* < 0.05 (Tukey’s test).

To elucidate the *in-vivo* effect of the isolated yeast on plant growth, we utilized *M. sativa* as our plant model. Our evaluation of the stress-tolerant yeast, L1 and L2, demonstrated their capacity to enhance alfalfa shoot growth and development. Statistical analyses revealed a significant impact of the tested strains on the shoot, root, and total biomass dry weight (Fig. 2). Inoculated plants showed higher shoot, root dry weight, and dry matter biomass (Fig. 2A). Inoculated plants exhibited notably higher shoot and root dry weight, as well as overall dry matter of biomass (Fig. 2A). Interestingly, there was no specific difference in the consortium’s effect compared to the application of L1 and L2 alone in terms of shoot, root, and total biomass, except in case of leaves number. Specifically, applying L1 resulted in a 53% increase in shoot dry weight, while L2 boosted it by 49% (Fig. 2A). Inoculation with mixed strains (L1 + L2) led to an 84% increase in root dry weight. Similarly, the shoot length, root length, and leaves number were significantly improved by applying yeasts (Fig. 2B). The shoot and root length were improved by 19% and 31% by the inoculation with *Rhodotorula* sp. L1 (Fig. 2B). The number of leaves was increased by 85% by applying the stress-tolerant yeast strains (L1 + L2).

#### Nitrogen, phosphorus, and potassium assimilation

The impact of chosen yeast inoculation on the acquisition of nutrients in *M. sativa* has been investigated (Table 2). The mineral acquisition showed varying distinctions between plants that were inoculated and noninoculated. A remarkable illustration of the impact of inoculation is the significant enhancement of nitrogen acquisition by 136% in plant that were inoculated with L1 + L2. Nevertheless, it is worth noting that phosphorus acquisition did not exhibit significant (*P* < 0.05) improvement by the biological treatment. Conversely, the inoculation with L2 notably enhanced the potassium acquisition, resulting in a significant improvement of 12%.

**Table 2.** Nitrogen, phosphorus, and potassium assimilation.

Treatment	N (mg/g)	P (mg/g)	K (mg/g)
C	3.55 ± (0.16)^c^	0.32 ± (0.03)^a^	21.89 ± (0.02)^d^
L1	7.84 ± (0.28)^b^	0.34 ± (0.02)^a^	23.89 ± (0.02)^b^
L2	7.65 ± (0.16)^b^	0.37 ± (0.01)^a^	24.59 ± (0.07)^a^
LM	8.4 ± (0.28)^a^	0.34 ± (0.02)^a^	22.32 ± (0.05)^c^

Means (±standard deviation) within the same column followed by different letters are significantly different at *P* < 0.05.

## Discussion

We hypothesized that in EEs, microorganisms have evolved and developed several tolerance mechanisms and distinctive PGP traits as a response to the selection pressure imposed by the harsh environment. Despite efforts, the understanding of yeast diversity and their functional capacities linked to extreme ecosystems remains insufficiently comprehended. We considered TM and MD to constitute a MEE for isolating unexamined-evolved non-*Saccharomyces* strains with unique, distinctive characteristics because of the high altitude, low oxygen level, atmospheric pressure, high temperature, and oligotrophic conditions.

In this study, we examined the growth-promoting attributes of yeast strains isolated for MEEs and their impact on the growth of *M. sativa*. Two yeasts, L1 and l2, were isolated from Morocco's highest peak and the largest desert dune , respectively. The results of the BLAST homology indicated a similarity between the red yeast L1 and *R. mucilaginosa*. In contrast, the extremophilic yeast L2 display a 99% sequence identity to *N. albida*. This is the first study reporting the isolation of *R. mucilaginosa* and *N. albida* from MEE. Our findings support earlier research that showed the wide prevalence of non-*Saccharomyces* strains from different EEs. For instance, *R. mucilaginosa* was isolated from EE, such as the Peninsula (Troncoso et al. 2017), Antarctic sea (Wang et al. 2019), oilfield (Derguine-Mecheri et al. 2021), and Sua pan (Loeto et al. 2021). Similarly, *N. albida* has been isolated from Antarctic soil samples (Białkowska et al. 2017), snow from the south pole (Hayward et al. 2021), and hypersaline coastal waters (Fotedar et al. 2022).

Yeast strains, being remarkably adaptable microorganisms, employ a variety of molecular and cellular mechanisms to endure and thrive in diverse EEs. The stress adaptation mechanisms in yeast are diverse and interconnected, often involving intricate signaling pathways, gene expression changes, and alterations in cellular physiology. In the face of elevated temperatures, yeasts adjust the concentration of saturated fatty acids (also reaching 30%–40%) occurring in lipids and maintain an optimal degree of fluidity (Buzzini et al. 2017). Indeed, yeast activates a robust heat shock response characterized by the induction of heat shock proteins (HSPs), which facilitate the proper folding and stabilization of proteins under thermal stress. The transcription factors Hsf1 and Msn2/Msn4 mediate the heat shock response. It involves the upregulation of several genes for HSPs that participate in trafficking and maturation, as well as the genes for the protein degradation machinery (Trott and Morano 2002, Mühlhofer et al. 2019). Conversely, under cold temperatures, yeast species exhibit a cold shock response, often involving the upregulation of specific cold shock proteins and physiological adaptations that decrease their growth rate and synthesize enzymes active at low temperatures and cryoprotective molecules to maintain membrane fluidity and cellular functions (Buzzini et al. 2017, Sanino et al. 2017). For instance, cold-adapted *R. diobovatum* possesses a notable attribute characterized by the elevated production of unsaturated fatty acids, thereby ensuring a heightened fluidity of the plasma membrane (Turk et al. 2011, Segal-Kischinevzky et al. 2022). In the case of *R. frigidialcoholis*, the response to low temperatures in the permafrost of the Antarctic dry valley is achieved through many mechanisms. These mechanisms include overexpression of the pentose phosphate pathway genes, increasing the production of carotenoids, sphingolipids, unsaturated fatty acid, and exopolysaccharides while coupled with a reduction in expression of growth, transcriptional, and translational machinery genes (Touchette et al. 2022). Yeast derived from arid habitats can endure the hydrological strain and generate aridity-enduring formations such as ascospores, teliospores, and chlamydospores, which sprout under favorable circumstances. At the same time, in their vegetative form, they can synthesize polysaccharide capsules that prevent desiccation (Buzzini et al. 2017). When confronted with nutrient deficiencies, yeast relies on sophisticated nutrient sensing and signaling pathways, such as the Target of Rapamycin pathway, to orchestrate adaptive responses. These responses may include the modulation of cellular growth rates and initiating autophagy, enabling yeast cells to recycle cellular components and sustain essential metabolic processes even in nutrient-limited environments (Conrad et al. 2014, Van Zeebroeck et al. 2021).

The findings of our investigation suggest that the isolated extremophilic yeast may be implicated in a broad spectrum of biological processes, offering potential application in the promotion of plant growth. The isolated yeast strains demonstrated salt and osmotic stress resistance and exhibited active characteristics associated with plant growth promotion. These characteristics include the ability to solubilize tricalcium and rock phosphate, solubilize potassium, produce ACC deaminase, and produce IAA and siderophores. In agreement with these findings, it has been documented that yeast strains isolated from various environment can display several PGP activities (Nassar et al. 2005, Amprayn et al. 2012, Nutaratat et al. 2014, Fu et al. 2016). Fu et al. (2016) have provided evidence that yeast strains exhibit a diverse spectrum of IAA synthesis, with concentrations ranging from 8 µg ml^−1^ (as observed in the case of *Kazachstania jiainicus* JYC361) to 610 µg ml^−1^ (seen in the case of *Aureobasidium pullulans* JYC104) depending on the species. Furthermore, it has been observed that *Candida tropicalis* HY possesses the capacity to dissolve phosphate to a concentration of 119 mg ml^−1^ (Amprayn et al. 2012). In this study, the extremophilic yeast strain, *R. mucilaginosa* L1, produces a significant amount of IAA (>100 µg/ml), which may have a deleterious effect on plant growth (Tapia-Vázquez et al. 2020); however, this strain shows a biotechnological potential for the production of IAA that is used in agriculture as a rooter. As demonstrated by Sen et al. (2019), the genome of *R. mucilaginosa* encodes genes associated with PGP, including auxin biosynthesis, cytokinin metabolism, abscisic acid and gibberellin biosynthesis, and jasmonic acid production. On the other hand, Sen et al. (2019) also highlighted the presence of specific genes coding for stress tolerance, especially for cold adaptation. This evidence supports to our hypothesis, which asserts that yeast has undergone evolutionary adaptations to develop mechanisms of tolerance in EEs. In our specific case, these tolerance mechanisms pertain to salinity and drought. In contrast, there may be limited specific information on *N. albida* regarding plant growth promotion traits due to the limited number of studies reporting on the plant growth promotion traits of the *Naganishia* genus.

The isolated extremophilic yeast has demonstrated considerable utility by effectively promoting the growth of *M. sativa*. Whether applied individually or in combination, these yeast species have significantly improved both shoot/root dry weight and number of leaves. Moreover, our research has revealed that these extremophilic yeasts possess the ability to enhance nutrient uptake, as evidenced by their increased assimilation of essential nutrients such as N, P, and K. Numerous studies have also substantiated the capacity of yeast strains to promote the growth of *Oryza sativa, Solanum lycopersicum*, and *Nicotiana benthamiana* (Amprayn et al. 2012, Thais et al. 2019, Fernandez-San Millan et al. 2020, Tapia-Vázquez et al. 2020). Fernandez-San Millan et al. (2020) demonstrated the effectiveness of applying *R. dairenensis* in enhancing the growth and development of *Nicotiana benthamiana*. Similarly, Tapia-Vázquez et al. (2020) have confirmed the ability of psychrophilic and psychrotolerant yeasts (specifically *Rhodotorula* sp. and *Naganishia* sp.) to promote *Solanum lycopersicum*. However, despite the extensive use of yeast as a biocontrol agent, research on the application of yeast as a biofertilizer has been relatively limited compared to bacteria, actinobacteria, fungi, and mycorrhizae. Consequently, there exists a knowledge gap regarding the potential of yeast as PGP agents and the specific mechanisms by which they facilitate plant growth. Nevertheless, there has been a recent surge in scientific interest to explore the PGP potential of yeast. This newfound attention is primarily driven by their ability to produce phytohormone compounds, solubilize inorganic phosphate, excrete siderophores, and possess ACC deaminase activity (Hamidi et al. 2020, Tapia-Vázquez et al. 2020, Ramya et al. 2021, Srinivasan et al. 2022).

## Conclusion

While the untapped potential of yeasts as agents for promoting plant growth remains unexploited, it is clear that yeast has a substantial opportunity to establish itself as an indispensable component of sustainable agriculture in the coming decades. In this investigation, we have unveiled the potential of two yeast strains that are not non-*Saccharomyces*. These strains, isolated from TM and MD, have displayed various PGP traits and demonstrated their ability to promote plant growth under greenhouse conditions. Expanding upon these discoveries and exploring the full potential of these two isolated strains, *Rhodotorula* sp. L1 and *Naganishia* sp. L2, is imperative in promoting plant growth under different stressed environments.

## Data Availability

The authors confirm that the data supporting the findings of this study are available within the article.orting the findings of this study are available within the article.
